# PRF-Solution in Large Sinus Membrane Perforation with Simultaneous Implant Placement-Micro CT and Histological Analysis

**DOI:** 10.3390/membranes11060438

**Published:** 2021-06-10

**Authors:** Horia Mihail Barbu, Stefania Andrada Iancu, Violeta Hancu, Daniel Referendaru, Joseph Nissan, Sarit Naishlos

**Affiliations:** 1 Head of Oral Implantology Department, Faculty of Dental Medicine, Titu Maiorescu University, 031593 Bucharest, Romania; horia.barbu@gmail.com; 2 European Centre of Oral Implantology, 011473 Bucharest, Romania; danreferendaru@gmail.com; 3 Department of Prosthodontics, Faculty of Dental Medicine, Titu Maiorescu University, 031593 Bucharest, Romania; 4 Titu Maiorescu Doctoral School of Dental Medicine, 040441 Bucharest, Romania; 5 Oral Health Department, Faculty of Dental Medicine, Titu Maiorescu University, 031593 Bucharest, Romania; 6 Oral Implantology Department, Faculty of Dental Medicine, Titu Maiorescu University, 031593 Bucharest, Romania; 7 Head of Oral-Rehabilitation Department, School of Dental Medicine, Tel-Aviv University, Tel-Aviv 6997801, Israel; nissandr@gmail.com; 8 Department of Pediatric Dentistry, School of Dental Medicine, Tel-Aviv University, Tel-Aviv 6997801, Israel; river554@gmail.com

**Keywords:** sinus floor augmentation, sinus graft infection, Schneiderian membrane perforation, sinus membrane suture, platelet rich fibrin (PRF), sinus mucocele, sinus complications

## Abstract

Background: The purpose of the study was to analyze the efficacy of platelet-rich fibrin (PRF) as a single augmentation material for complicated cases of maxillary sinus floor elevation, resulting from membrane perforation or previous infections. Methods: Implant insertion in the posterior region of the maxilla was simultaneously performed with maxillary sinus floor augmentation. Schneiderian membrane elevation can be accompanied by extremely serious sinus membrane perforation, due to accidental tearing or intended incision for mucocele removal. PRFs were placed in the sinus cavity both for membrane sealing and sinus floor grafting. Radiological, histological and micro-CT analyses were performed. Implant survival was assessed every 6 months for 1 to 4 years, with a mean follow up of 1.8 years, after prosthetic loading. Radiological examinations were performed on CBCT at 9 and 12 and 36 months postoperatively and revealed improved degrees of radiopacity. Results: 19 implants were simultaneously placed in the course of nine maxillary sinus floor augmentation surgeries, with successful outcomes in terms of bone grafting and implant integration. New bone formation was evidenced 12 months postoperatively on radiological examination, micro-CT analysis, and histological analysis of a harvested bone segment from the augmented maxillary sinus. The mean gain in bone height of the sinus floor augmentation was 6.43 mm, with a maximum of 9 mm. The mean amount of vital bone obtained from histologic assessment was 52.30%, while bone volume/tissue volume ratio in micro-CT 3D had a mean of 50.32%. Conclusions: PRF may be considered as an alternative treatment for a single surgery of sinus augmentation with simultaneous implant placement, even in complicated cases with significant sinus membrane tearing.

## 1. Introduction

Modern implant dentistry is tightly connected to bone augmentation procedures, especially when implant insertion follows a prosthetic planning concept. Among numerous bone grafting techniques, one of the most frequently used procedures is maxillary sinus floor augmentation [1]. Surgical techniques vary in the way the sinus cavity is accessed (lateral or crestal approach) and the type of grafting material placed into the cavity [2]. After the elevation of the sinus membrane, the cavity can be filled up with different types of bone grafting material, such as xenograft, autograft, alloplast, porous titanium particles, PRF, or a combination of these materials [3,4,5]. When used correctly, all materials can offer viable results for the creation of sufficient hard tissue for implants with adequate length and diameter.

No matter what technique is used for the elevation of the Schneiderian membrane or what grafting materials we use, the purpose is to achieve an appropriate implant site, specifically to increase the bone volume, through the formation of good quality bone.

Maxillary sinus augmentation might involve common surgical intra- and postoperative complications, or specific related obstacles that the surgeon has to deal with. Of great importance is the risk of intraoperative bleeding due to improper manipulation of or failure to observe the antral alveolar artery using computed tomography [6].

The highest incidence is the perforation of the Schneiderian membrane during its elevation, which can entail further complications, such as graft infection, implant failure, and sinusitis [7,8]. Large sinus membrane perforations increase surgical morbidity and may even require changing a previously established single-staged treatment plan into multiple stages, to separate the grafting procedure from the implant placement.

PRF (plasma-rich fibrin) was firstly reported by Choukroun in 2001, and ever since, the concentrate has found wide application in oral surgery [9,10,11]. It is easy to handle and to procure, through centrifugation from the venous blood of the patient [10,12,13]. Due to its biological properties, PRF is feasible for sealing Schneiderian membrane perforation, optimizing the healing process, and even for use as the sole material for new bone formation in the maxillary sinus [14,15]. The biological attributes of PRF membranes include the impactation of numerous viable blood cells, but also the capacity for cell proliferation and cell adhesion of PRFs in healing process and overall tissue engineering [16]. The purpose of this study was to develop a surgical protocol for overcoming the complications related to extremely large perforations caused by improper membrane manipulation, cases where the sinus membrane is extremely thin and fragile, and when the membrane has been intentionally cut in order to remove a retention cyst. At the same time, we analyze the benefits and the disadvantages of PRF when used as a single grafting material in nine nine complex sinus augmentation procedures with Schneiderian membrane perforation and simultaneous implant insertion.

## 2. Material and Methods

### 2.1. Analysis Design and Case Selection

This retrospective and descriptive study was designed as a case series based on nine surgeries, which were analyzed on the basis of intra-operative photos and cone beam computed tomographies (CBCTs) from a digital database of a private dental clinic, located in Bucharest, Romania, called the European Centre of Implantology. The information was completed by histological evaluation and micro-CT analysis of the newly formed bone, performed at Ankara University, Turkey.

The augmentation procedures were performed by the same operator, who followed the same protocol. The patients included in the study approved their participation in the clinical trial and provided informed consent to this effect. This retrospective study followed the ethical principles of the Helsinki Declaration involving human subjects, having the purpose of improving a therapeutic intervention, while simultaneously protecting the rights of the patients.

This research followed the STROBE guidelines and was performed in accordance with the ethical protocol that ensures and promotes the rights and health of patients. The Institutional Review Board obtained the approval (No. UTM03FEB20-MD20) from the ethical committee at Titu Maiorescu University, Bucharest, Romania. The potentially eligible patients were selected according to the following, cumulative, variables of interest: need for sinus floor augmentation by lateral approach → simultaneous implant placement → very large Schneiderian membrane perforation → sinus membrane sealing performed with suture, collagen wound dressing membrane and/or PRFs → PRFs as a single material placed for sinus floor augmentation. The inclusion criteria for the patients are listed in Table 1.

### 2.2. Surgical Phase

Local anesthesia was performed with articaine hydrochloride (Ubistesin Forte, 3 M ESPE, Minneapolis, MN, USA) and a full-thickness flap was elevated to allow access to the anterior wall of the maxillary sinus. The osteotomy was performed using an ultrasound surgical device (Piezomed by W&H, Bürmoos, Austria), with constant saline irrigation. After complete delimitation of the bony window and its removal, gentle elevation of the Schneiderian membrane was performed. Due to membrane friability, the presence of sinus septa, or intentional membrane incision for removing a retention cyst, a tear/perforation resulted that was larger than 15 mm.

**Figure 1:**
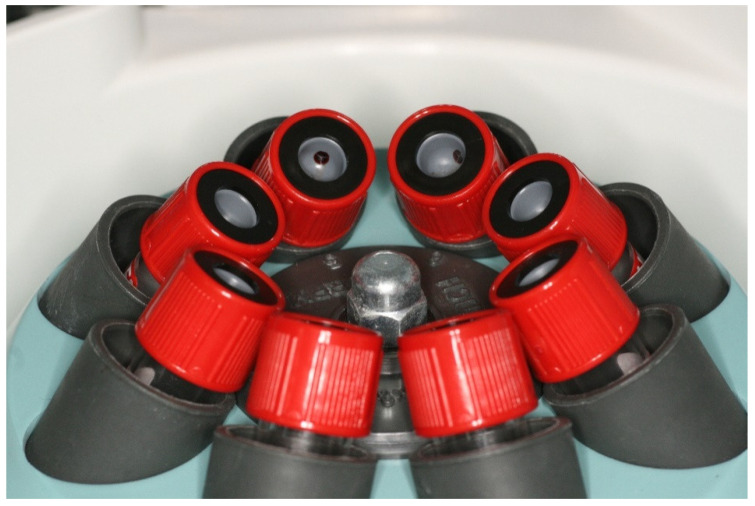
Single-use vacutainers filled with venous blood prepared for centrifugation.

**Figure 2:**
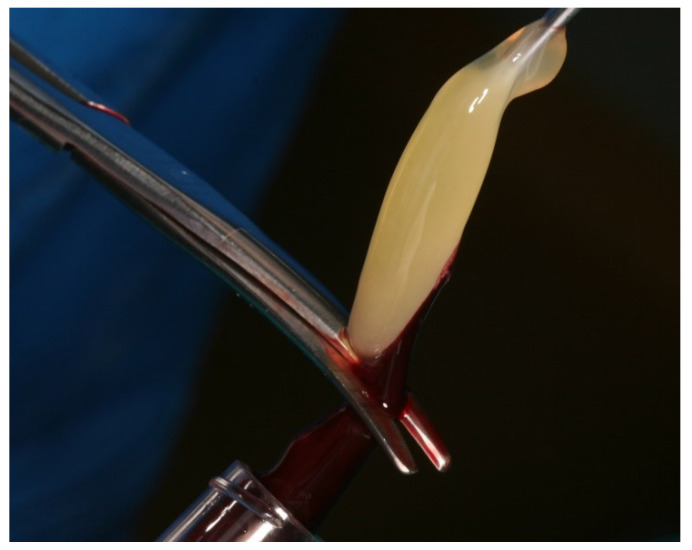
Separation of the PRF fibrin product from the red blood clot.

PRF clots were produced intra-operatively following a standard protocol: venous blood of the patients was collected in 9 mL vacutainers without clot activator or gel separator. Blood collection was performed with pre-attached, pre-fabricated sets (Vacutainer^®^ Safety-Lok, Becton, Dickinson & C., Franklin Lakes, NJ, USA). Immediately after blood collection, the vacutainers were centrifuged at 2700 rpm for 12 min (Figure 1). At the end of the centrifugation process, there were three layers in the tubes, represented by the red blood cells at the bottom, the fibrin and red clot in the middle, and serum at the top. Using an atraumatic tweezer, the fibrin matrix was extracted from the vacutainers and separated from the red clot with a scalpel or scissors (Figure 2).

**Figure 3:**
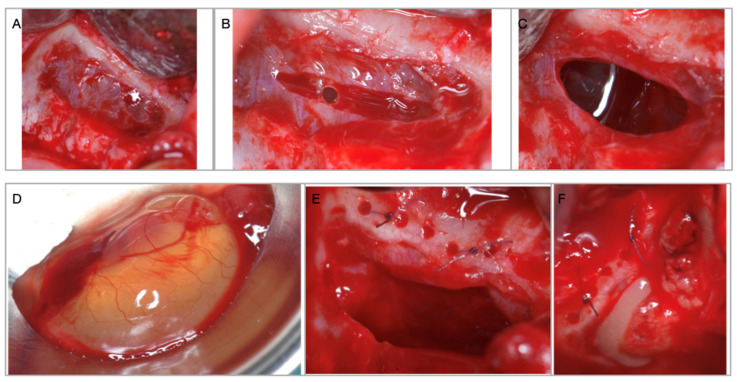
(**A**) Access window through the anterior wall of the maxillary sinus with intact Schneiderian membrane. (**B**) Deliberate incision in the sinus membrane for mucocele removal. (**C**) Size of the perforation necessary for cyst removal. (**D**) Maxillary sinus mucocele. (**E**) Schneiderian membrane perforation is sutured to the superior bony edge. (**F**) Sinus floor augmentation performed with PRFs after the implants are inserted.

While the PRF were prepared, the surgeon sealed, by suturing, the perforations. Due to the size of the perforations or increased membrane friability, tight sealing was not possible in all cases, and a configuration like a “net” was implemented at the roof of the cavity, subsequently resulting in multiple, but smaller, perforations. This “net” was then fixed by suture to the bone, on the upper part of the window, through 3–4 holes that were prepared with a 1 mm drill (Figure 3E).

Suture was always accompanied by PRF membrane coverage. The membranes were obtained through slight, homogenous compression of the fibrin clot in the specially designed PRF metal case (Medco Instruments, Hickory Hills, IL, USA). In two cases, where the PRF membranes were not large enough to completely seal the perforations, a quickly resorbing (10–14 days) large wound dressing collagen membrane (Collatape, Zimmer Biomet, Warsaw, IN, USA) was placed inside the cavity, over the sutured membrane.

Implant osteotomy was then performed and 1 to 2 implants were placed and stabilized in the residual subantral bone. The entire space between the implants, the sinus floor and the elevated Schneiderian membrane was filled up with PRF clots (Figure 3F). The access window was protected in 8 cases by a low resorption pericardium membrane (Copios, Zimmer Biomet, Warsaw, IN, USA), and in one case by the bony window which was placed back, fixed with osteosynthesis screws.

### 2.3. Postoperative Protocol

All patients were prescribed medications and were given written indications to follow after the surgery. One day before the surgery, and continuing for the next two days, they were all administered steroidal anti-inflammatory drugs (dexamethasone, 8 mg). For 7 days, they were prescribed antibiotics, 1 g every 12 h of amoxicillin combined with clavulanic acid. They were instructed to avoid sneezing with the mouth closed, straw drinking and not to blow the nose.

All patients were recalled for postoperative control 48 h after the surgery, and then after 7 days for suture removal, and every 4 weeks until the new-formed bone was visible on CBCT. Implant uncovering was performed 9 to 12 months later, and their initial loading was performed with temporary restorations.

### 2.4. Radiological Analysis

Cone beam computed topographies (CBCTs) were analyzed by the same operator in a single pre-augmentation site. The initial bone height was measured on a CBCT in the area where it was planned to place the implant. On the CBCT performed 12 months after the surgery, the same operator measured the bone height gained.

### 2.5. Histologic Processing

**Figure 4:**
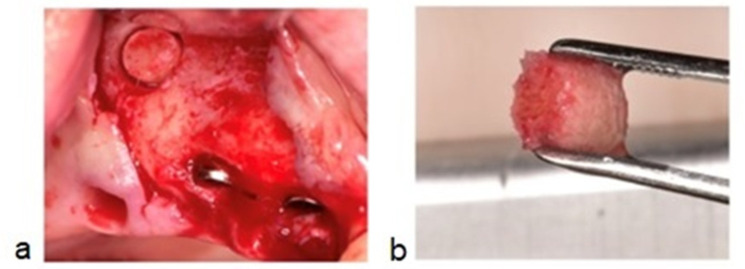
(**a**) (Case no.4) Bone osteotomy performed between the second and third implant, above the residual bone height, 8 10 mm apically from the edentulous ridge. (**b**) Cylindrical biopsy core of 3–4 mm, equivalent to the drilling depth.

A trephine drill (Devemed GmbH, Tutlingen, Germany) was used to harvest bone, 3–4 mm deep, between the last two implants, at 8–10 mm apically, above the residual bone height, perpendicular to the anterior wall of the sinus, for histological analysis (Figure 4). After fixation in formaldehyde solution 4%, the bone specimens were rinsed in water, dehydrated in increasing concentrations of ethanol, infiltrated, and embedded in methyl methacrylate. Un-decalcified longitudinal sections (300–350 μm) were prepared using a diamond saw microtome technique (Exakt 300 CL, Exakt Apparatbau, Norderstad, Germany). These sections representing the full length of bone were additionally ground (40 μm) and polished (Exakt 400 CS, Exakt Apparatbau, Norderstad, Germany). Staining was performed with Toluidine Blue. Images were obtained with the Microscope Olympus^®^ CX41 (Tokyo, Japan) and digitized with a video camera (Olympus^®^ DP 25, Tokyo, Japan).

### 2.6. Point-Counting Method

**Figure 5:**
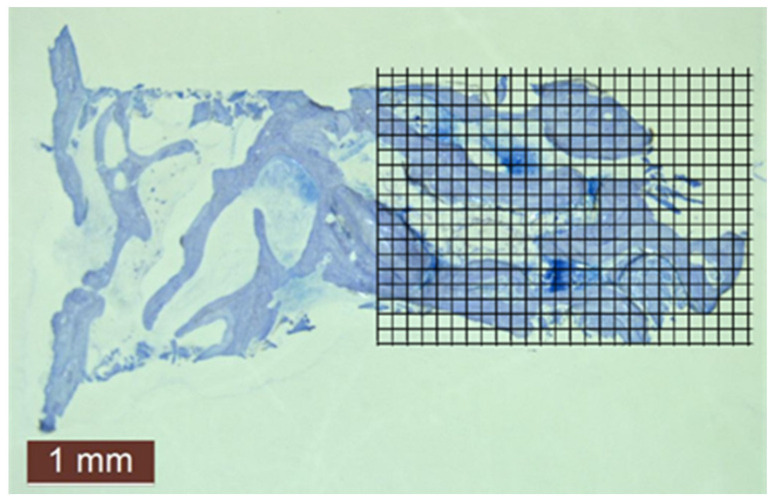
Histomorphometric evaluation of new bone amount by point counting method in case no. 4.

A grid of 20 × 20-pixel squares was placed on the pictures taken with 10× magnification from histological sections (Figure 5). The points coming to the bone surfaces were counted in an area that was standard for each image, where a total of 390 squares (26 × 15) were included on each grid.

The points counted for each picture were divided by the total number of points, and their percentages were calculated by two independent examiners (bone point counts/total point counts (390) × 100).

### 2.7. Micro-CT Analysis

The CTan software provided by the manufacturer was used to obtained reconstructed bone biopsy images and volumetric analysis measurements in NRecon software. In the CTan program, each sample was analyzed separately. Following reconstruction, adaptive inter-polarization was applied on bone biopsy specimens (after histologic processing in formaldehyde), and ROIs (study areas) were determined for volume measurement in the axial images of bone biopsy specimens. An appropriate threshold setting was implemented to separate normal bone tissue from other tissues. To eliminate soft tissues, the lowest grey scale value was adjusted, and segmentation was performed. Artefact spots on the ROI were removed from the area. 3D bone volumes were calculated for each sample using CTVol v.2.2.1 software (BrukermikroBT).

## 3. Results

The analysis focused on nine surgeries, performed on five male (56%) and four female (44%) patients, aged between 36 and 64 years, with a mean age of 49.44 years. All sinus floor augmentation surgeries were performed between 2014 and 2018. For each case, the surgical protocol, as well as the radiological analysis, were similar.

All maxillary sinus grafting procedures were performed with simultaneous implant placement. A total of 19 implants were inserted, differing in length, diameter and brand, as follows: two ARDS implants (4.2 mm in diameter and 11.5 mm in length), eight Zimmer Biomet implants (3.7 mm, 4.1 mm and 4.7 mm in diameter and 13 mm and 16 mm in length), six ADIN implants (3.75 mm and 4.2 mm in diameter and 13 mm and 16 mm in length), and three IBS implants (4.5 mm in diameter and 13 mm in length).

The minimum initial subantral bone height was 2.6 mm, which was measured in a 64-year-old male patient, for whom the post-augmentation measurements revealed a bone gain in height of 7.05 mm. The maximum postoperative subantral bone gain was 9 mm, measured in a 39-year-old female patient (Table 2). The mean initial bone height was 4.48 mm, with 1.45 mm standard deviation (SD), while the mean gain in bone height was 6.43 mm, with 1.88 mm SD. When calculating the t-test, the values describing the difference between the initial and the postoperative bone possessed statistical significance (*p* < 0.005). Non-parametric statistics was performed to assess the relationship between the augmented bone and the implants. As a result, there was no correlation between the length of the implant used in the study and the gain in bone height (*r* = 0.139).

Large Schneiderian membrane perforations (greater than 15 mm) occurred in all nine cases (100%) and were sealed by membrane suture and PRF membranes (seven cases, representing 78%), or, fast resorption (10–14 days) rate collagen wound dressing membrane coverage (22%, equivalent to two cases).

**Figure 6:**
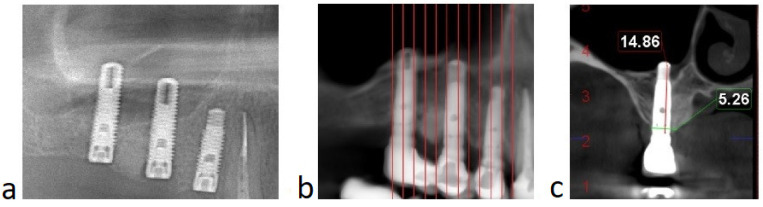
(Case no. 4) Radiological aspect of the sinus floor augmentation performed with PRFs, with different degrees of radiodensity: immediately after the surgery (**a**); and 12 months after the procedure (**b**). Specific image of sinus augmentation with PRF where the tip of the implant (1–1.5 mm) has formed no bone, due to Schneiderian membrane pressure during the new bone formation process (**c**).

All patients from the group presented successful outcomes with respect to implant integration and sinus augmentation procedures. They exhibited reduced, or even no, pain after surgery, and minimal discomfort due to edema. Clinical evaluation of the surgical site revealed good healing, with normal color and texture of the soft tissue. Signs of infection, dehiscence or inflammation were absent for all nine patients (100%). Implant uncovering demonstrated good osseointegration, with no mobility and no vertical bone loss. Implant survival was assessed every 6 months for 1 to 4 years, with a mean follow up of 1.8 years, after the prosthetic loading. Radiological examinations were performed on CBCT at 9 and 12 and 36 months postoperatively and revealed improved degrees of radiopacity from the first to the last CBCT. The first radiological examination showed a homogenous radiopacity of the PRF membranes with a net delimitation from the sinus cavity. Months later, the implants appeared to be surrounded by a dense bony-like structure, with no peri-implant radiolucency (Figure 6). The apices of the implants, as well as 1 mm below the tip of the implant, showed no bone formation due to the fact that the membrane is leaning on it.

**Figure 7:**
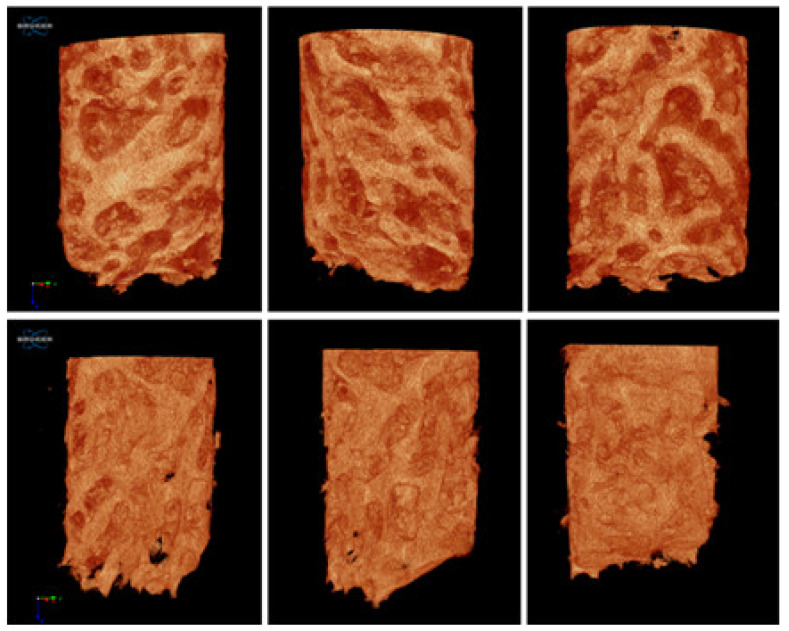
Micro-CT reconstruction of the harvested bone. In case no. 4.

The amount of vital bone obtained from histological assessment shows a mean of 52.30%, while the bone volume/tissue volume (BV/TV) ratio in micro-CT 3D had a mean of 50.32% (Figure 7).

## 4. Discussion

Research into medical technologies and procedures has the purpose to simplifying the practitioner’s work and increasing the patient’s comfort and quality of life, reducing morbidity and promoting dental health. The expected results of the treatment should be directed by the surgical technique, along with the necessary materials, in a single stage, or, if necessary, when complications occur, in separate procedures.

Bone grafting is considered to have been successful when the histological and mechanical properties resemble the native bone of the patient. The result should allow good primary stability of the implant and should be stable over time, ensuring long term survival. The variety of bone substitute materials available for sinus floor augmentation procedures should offer different possibilities for particular clinical cases. Despite the numerous studies emphasizing a specific bone grafting material, sometimes, a surgeon has the best results with the techniques and materials that they have the greatest mastery of.

Many generations of autologous platelet concentrates (APCs) have been developed to improve local tissue healing and regeneration under oral conditions or subsequent to surgical wounds. Examples of these preparations include autologous platelet gel (APG), known as plasma rich in growth factors (PRGF), platelet-rich plasma (PRP) and platelet-rich fibrin (PRF) [17]. In comparison to PRF, PRGF and PRP have a more complex preparation process (two stages of centrifugation and additional use of coagulation factors, CaCl_2_ or bovine thrombin) [17]. Furthermore, PRP and PRGF have a limited potential for the bone regeneration process due to their short periods of growth factor release, as well as their weak fibrin matrix [17].

Platelet-rich fibrin is a second-generation APC, obtained from the patient’s blood, that is inexpensive and easy to produce through centrifugation in collection tubes without anticoagulant or any kind of biochemical manipulation [10,12,17,18,19]. The coagulation pathway occurs during centrifugation and separates the blood into three different layers: serum at the top, the PRF clot in the middle and the red blood cells in the lower layer [10,12,18]. The middle PRF clot is composed of fibrin and fibrin matrix concentrated in growth factors [10,18].

To PRF clot is attributed two main properties of the sinus floor bone augmentation process:

A. From a mechanical point of view, the PRF provides, as a result of its volume, a relative scaffold (because it will be resorbed after a few days) between the implants and the sinus membrane for cell migration during healing and bone formation [12]. Thus, the PRF clot, as formed in the collection tube, is preferable in the sinus cavity, rather than PRF membranes [9]. Another important principle is the elevation of the sinus membrane and its long-term maintenance in a stable position at the desired height from the sinus floor [9]. Some authors have reported the fact that sinus bone formation does not require additional biomaterials [20]. Chen et al. associated bone formation with the maintenance of the required space between the membrane and the bone cavity, followed by blood clot formation and osteoblast migration from the sinus periosteum [20]. A study performed in 2016 concluded that residual bone heights >4 mm allowed bone formation in the sinus cavity without any grafting materials [21].

Because of the rapid resorption rate of the PRF compared to the bone formation process, PRFs alone are not suitable for maxillary sinus augmentation without the presence of the implants [9]. Simultaneous implant placement should be considered, in order for the implant apices to maintain an elevated Schneiderian membrane [9]. As a result of the membrane, following PRF resorption, leaning on the tip of the implants, that area (1.5 mm) will not form bone; for example, a 13 mm implant will only form bone in the first 11.5 mm [22]. Compared to other bone grafting materials, the volume of the future augmentation site is preserved by the particulate materials with no or minimal resorption [9]. Thus, PRF as the sole material for sinus floor augmentation is indicated only in cases where the residual subantral bone height and density allows primary implant stability.

The bone-to-volume ratio (BV/TV) resulting from the micro-CT evaluation represents the amount of mineralized bone volume in the entire segment of bone biopsy. The trabecular bone microarchitecture in this study reveals a denser bone structure in comparison to other studies that performed micro-CT analysis 12 months after sinus floor elevation. Lundgren et al. concluded a mean bone volume fraction of 48% +/− 10% 12 months after sinus floor augmentation surgeries with autologous bone particles from the mandible [23].

B. The immunohistological benefits of the PRF consist of the abundance of leukocyte cytokines and red blood cells, which in conjunction with the fibrin clot, stimulate wound healing through the slow release of growth factors [17,19]. For example, platelet-derived growth factors (PDGF), as well as transforming growth factor-β (TGF-β), vascular endothelial growth factor (VEGF), insulin-like growth factor-1 (ILGF-1) and epidermal-growth factor (EGF) released by PRF, have a therapeutic function in bone formation and remodeling [12,17,24,25]. These growth factors induce chemotactic activity, and promote cellular differentiation and proliferation [17,19].

The different regenerative capacities among different ACPs is determined by the time required for fibrin polymerization, which determines the release period of the growth factors. PRF slowly releases growth factors, while PRGF has a short period in which release of growth factors takes place [17].

With different relative centrifugation forces (RCFs), distinctive PRF products have been described in the literature. Choukroun et al. produced the widely known Leukocyte-PRF (L-PRF). Lower RCFs produce an improved product with a higher concentration and better distribution of cytokines and leukocytes. Thus, reduced centrifugation forces lead to the valuable slow, constant release of growth factors [17]. Modifications with respect to the time and speed of the centrifugation process during preparation of the PRFs is meant to increase the concentration of macrophages and leukocytes, which also have important contribution in host defense [19].

These biological materials have received wide application in medical procedures such as facial plastic surgery, sinus floor augmentation, treatment of exposed furcation, gingival recession, and intrabony defects [17].

Common intraoperative complications as a result of sinus membrane elevation, or its perforation, are correlated with increased morbidity. A review of 12 studies performed in 2016 revealed an incidence of 23.5%, ranging from 3.6% to 41.8%, for membrane perforations [7]. Correct management of perforations tends to unveil different implant survival rates when comparing cases with intact and perforated Schneiderian membranes [7,26,27,28,29].

Very large (>15 mm), or multiple large, sinus membrane perforations sometimes change the treatment plan from a single surgery, with simultaneous grafting and implant placement, to a staged strategic approach [7,30]. Specifically, for grafting materials with non-autogenous origins, the risk of infection increases, because the recipient site should possess good healing potential in terms of vascularization, cell migration and proliferation, in order to incorporate the bone graft and the implants, and also to heal the supplementary wound resulting from membrane perforation. The increased morbidity is the major reason to delay the implant insertion.

In addition, migration of xenograft or alloplast grafting materials into the sinus cavity is associated with a higher risk of sinusitis [9]. At the opposite pole, PRF is associated with a lower risk of infection if it migrates through the perforation into the maxillary sinus [9].

Improper Schneiderian membrane sealing exposes the bone substitute material to the specific microbial flora present in the maxillary sinus [30]. A compromised sterility of alloplast or xenograft leads to infection, affecting both the augmentation and the implants [30]. The PRFs in direct contact with the non-sterile environment of the maxillary sinus act as a barrier membrane with respect to the unsealed perforation, speeding up the healing of the perforation. In addition to the release of the growth factors, PRF is also attributed with the role of fighting bacterial infections [31].

PRF is a low-cost alternative for sinus membrane sealing because of its “sticky” consistency, being a natural fibrin scaffold, which does not induce a foreign body reaction, as is the case with collagen membranes [31,32]. Some authors have recommended a double-layering of PRF to treat cases of significant Schneiderian membrane perforation [31].

In sinus floor augmentation, PRF can be used as a single material or combined with other bone grafting substitutes [18,31,33]. The combination of PRF clot fragments with other grafting materials accelerates bone regeneration, but increases the risks of infection in cases with multiple or large instances of tearing of the sinus membrane. As a general rule, delayed implantation or sinuses wider than 10 mm are not indicated for PRF as a unique grafting material [31].

Important disadvantages to be mentioned include the long-term period required for new bone formation and the unpredictable results of future augmented height, resulting from rapid PRF degradation [13].

One of the study’s limitations is the sample size. The small number of cases included in the study group makes it difficult to generalize the results with this specific surgical technique and material. Thus, in the absence of a larger sample size, the statistical analysis is not representative. It is not possible to make an accurate population distribution for the group of patients with whom the results are related.

Sources of bias are represented by the different Schneiderian membrane sealing techniques. In two cases, membrane sealing did not follow the same protocol, being dependent on perforation size, location, and the friability or elasticity of the sinus membrane. Although the entire study was based on the analysis of autologous materials used in surgery, an exception was made in the case of very large sinus membrane perforations, which were sealed with suture, PRFs and collagen membrane.

When compared to other foreign biomaterials brought to the recipient site (e.g., xenograft, alloplast), autologous materials are associated with reduced costs and no risk of infection [9]. The PRF product ensures accelerated angiogenesis, high biocompatibility, and faster wound healing [19]. It makes a substantial contribution towards the bone maturation process and hemostasis [19].

## 5. Conclusions

Within the limitations of this study, we can conclude that, using the PRF as a sole graft material, at the same time as implant placement, is indicated to simplify treatment from requiring multiple stages to being a one-stage surgery for sinus augmentation, even in complicated cases with significant tearing of the sinus membrane.

## Figures and Tables

**Table 1:** Characteristics of the patients included in the research.

Inclusion Criteria	Exclusion Criteria
Large Schneiderian membrane perforations (>15 mm) that cannot be sealed with any type of membrane by itself	Other materials than PRFs placed for sinus floor augmentation
CBCT examinations performed preoperatively and postoperatively	Patients who did not give their consent to harvest a bone core for histological analysis
Bone width (minimum 5.5 mm) and bone height (minimum 3 mm) to ensure primary implant stability	Patients who underwent a separated surgery for implant placement
Membrane suturing was possible to for a complete sealing or at least to obtain a “net”	Bone width less than 5.5 and bone height more than 7 mm

**Table 2:** Initial and 12 months postsurgical bone height, measured on CBCT sections in the same surgical implant site.

	1	2	3	4	5	6	7	8	9
Initial bone height (mm)	4.00	3.00	5	5.38	5.38	3.25	4.5	7.2	2.6
Bone gain in height (mm)	7.5	9	3	8.72	4.82	5.75	6	6.04	7.05

## Data Availability

The data presented in this study are available on request from the corresponding author. The data are not publicly available due to privacy policy.
